# Percutaneous Needle Electrolysis Reverses Neurographic Signs of Nerve Entrapment by Induced Fibrosis in Mice

**DOI:** 10.1155/2020/6615563

**Published:** 2020-12-22

**Authors:** R. Margalef, F. Valera-Garrido, F. Minaya-Muñoz, M. Bosque, N. Ortiz, M. M. Santafe

**Affiliations:** ^1^Unit of Histology and Neurobiology, Department of Basic Medical Sciences, Faculty of Medicine and Health Sciences, Rovira i Virgili University, Carrer St. Llorenc, No. 21, 43201 Reus, Spain; ^2^MVClinic Institute, Madrid, Spain; ^3^Department of Physical Therapy, CEU San Pablo University, Madrid, Spain; ^4^Getafe CF, Madrid, Spain; ^5^Neurology Department, Hospital Universitari Sant Joan de Reus, 43202 Reus, Spain

## Abstract

Nerve entrapments such as carpal tunnel syndrome are the most common mononeuropathies. The lesional mechanism includes a scarring reaction that causes a vascular compromise. The most effective treatment is surgery, which consists of removing the scarred area, thus reverting the vascular impairment. In the present study, a more conservative therapeutic approach has been undertaken to release the nerve by means of galvanic current (GC) applied with a needle: percutaneous needle electrolysis (PNE). For this purpose, a mouse model of sciatic nerve entrapment has been created using albumin coagulated by glutaraldehyde (albumin 35% and glutaraldehyde 2% volume applied, 10 *μ*l). After two weeks, a fibrous reaction was obtained which entrapped the nerve to the extent of causing atrophy of the leg musculature (14.7%, *P* < 0.05 compared to the control leg). Ultrasound imaging confirmed that the model's image was compatible with that of nerve entrapment in patients. To quantify the degree of entrapment, nerve conduction recordings were made. The amplitude (peak-to-peak) of the compound muscle action potential (CMAPs) decreased by 32.2% (*P* < 0.05), and the proximal latency increases by 17.7% (*P* < 0.05, in both cases). In order to release the sciatic nerve, PNE was applied (1.5 mA for 3 seconds and 3 repetitions; 1.5/3/3) by means of a solid needle in the immediacy of perineural fibrosis before and 5 minutes after the application of GC, and the proximal latency shows a decrease of 16% (*P* < 0.05). The recovery of CMAPs amplitude was about 48.7% (*P* < 0.05). Three weeks later, the CMAPs amplitude was almost completely recovered (94.64%). Therefore, with the application of GC by means of a solid needle, the sciatic nerve was definitively released from its fibrous entrapment.

## 1. Introduction

Mononeuropathies by entrapment are the most frequent neuropathies. Among these, the most common is carpal tunnel syndrome (CTS). This syndrome is prevalent in 4-5% of the population and is especially prevalent in the 40–60-year-old population. The prevalence rate is higher in women (9.2%) than in men (6%), between the ages of 45 and 60 years [1]. The most common clinical symptoms are paresthesia, pain, and muscle weakness in the area of nerve innervation [2]. Over time, this weakness can develop into severe atrophy.

The main mechanisms involved in these mononeuropathies include increased pressure on the microcirculation of the nerve, compression of the connective tissue surrounding the nerve, and hypertrophy of the synovial tissue [3]. Increased pressure on the microcirculation of the nerve and the ischemia of the nerve cause a decrease in nerve function at this level [4]. Secondary to increased intraneural pressure, there are also alterations in the axonal myelin sheath including axonal damage [5]. Nerve demyelination develops at the compression site and can then extend to the entire segment. A nerve conduction block will occur, and if compression persists, it may eventually interrupt blood flow to the endoneural capillary system [6], which leads to alterations in the blood nerve barrier and the development of endoneural edema. This initiates a vicious circle consisting of venous congestion, ischemia, and local metabolic alterations [6], and axonal degeneration, attraction and activation of macrophages, release of inflammatory cytokines, nitric oxide, and development of chemical neuritis are consequences of this vicious cycle if it remains for a considerable time.

Synovial tissue hypertrophy of tendons can increase pressure in the carpal tunnel and lead to the development of CTS [7]. Tenosynovitis is a factor closely related to the development of idiopathic CTS [7]. This has been confirmed by the presence of increased expression of prostaglandin E2 and VEGF in the synovial biopsy tissue of patients with CTS [8]. In response to this injury, there is an increase in fibroblast density, collagen fiber size, vascular proliferation, and type III collagen in the synovial connective tissue [9].

The treatment of this form of mononeuropathy can be conservative or surgical. Although both approaches have been shown to have benefits, surgical treatment has more lasting effects [10, 11]. However, surgical treatment is not exempt of complications [12] such as generation of a scar with adhesions, relapses, neurovascular injury, wound complications, deep pain at the base of the thenar eminence and wrist, and reduced strength. For this reason, therapeutic alternatives with minimal associated adverse effects are being explored. In this sense, Lemke et al. [13] have created an animal model that mimics the side effects of nerve surgery in humans through a chemically induced fibrous perineural reaction. This model shows delayed nerve conduction due to scars visible by light microscopy. The present study is based on this work.

The use of percutaneous needle electrolysis (PNE) has become popular in recent years. It consists of the application of galvanic current through a solid metal needle [14]. PNE creates an inflammatory reaction in the treated tissue due to the formation of inflammasomes that release IL-1b via the inflammatory caspase and caspase-1 [15]. In addition, the GC generates a markedly basic pH at the tip of the needle that can hydrolyze scar tissue [16]. The use of PNE has extended to pathologies related to nerve compression. Its action combines the mechanical effect of a solid needle and the galvanic current as a disruptive mechanism for the connective tissue, thus freeing the nerve from the pressure of the surrounding tissue, improving the patient's symptoms. However, the use of PNE has not been adequately evaluated for releasing scarred nerves. In this work, an animal model of a nerve entrapment caused by a provoked fibrosis has been created, and the release of the same is evaluated with galvanic current.

## 2. Materials and Methods

Experiments were performed on young adult male C57BL/6 mice (45–50 days postnatal, *n* = 70; Charles River, L'Arbresle, France). All experiments were carried out in the vivarium and in the neurophysiology laboratory of the Faculty of Medicine of the Rovira i Virgili University. Animals were housed in standard MAKROLON^R^ cages (27 × 27 × 14 cm^3^), and two animals were distributed for each cage. The temperature was maintained at 20–22°C by an electronic thermostat, and relative humidity was fixed at 60–70%. The circadian rhythms were 12 hours of white neon light and 12 hours of darkness. Feeding and hydration of animals was ad libitum, based on the VRF-1 mouse supplied by PANLAB (Spain) and regular tap water. The mice were cared for in accordance with the guidelines of the European Community's Council Directive (2010/63/EU) and the Spanish Royal Decree 53/2013 for the humane treatment of laboratory animals. This study was approved by the Ethics Committee of the Rovira i Virgili University.

### 2.1. Perinervous Scar Reaction Model

A model of perineural fibrosis in mice was developed from previous work in rats [13]. A solution of albumin (Bovine Serum Albumin, BSA) and glutaraldehyde (Glt) named glutaraldehyde glue (GG) (BSA 35% and Glt 2%) was used. For this purpose, several volumes of GG and periods were studied, distributed in five groups. Group 1, a volume of GG 50 *μ*l was administered and evaluated at 2 weeks. Group 2, a volume of GG 20 *μ*l was administered and evaluated after 2 weeks. In group 3, a volume of GG 10 *μ*l was administered and evaluated 1 week later. For group 4, a volume of GG 10 *μ*l was administered and evaluated at 2 weeks. Finally, for group 5, a volume of GG 10 *μ*l was administered and evaluated at 3 weeks.

**Figure 1:**
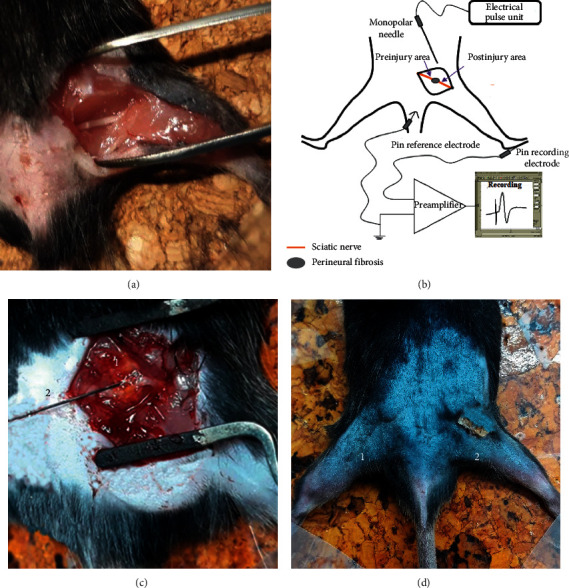
Perineural fibrosis procedure and application of percutaneous needle electrolysis (PNE). (a) Sciatic nerve exposed before treatment to generate perineural fibrosis. (b) Electrophysiological recording procedure diagram. (c) Application of PNE in the area with perineural fibrosis [1] with a solid needle [2]. (d) Atrophy of the posterior muscles, comparing the normal size of the mouse paw [1] with the treated paw [2].

The sciatic nerve of mice has been chosen as the animal model for CTS, since its caliber is very similar to that of the median nerve when passing through the carpal tunnel. After anesthetizing the animal and shaving the lumbar area and hind legs, a deep incision was made to expose the sciatic nerve (Figure 1(a)). The sciatic nerve was covered with the GG solution at a distance of 3 mm from the spine and, immediately afterward, the skin was sutured.

### 2.2. Neurographies

The equipment used to carry out the registrations consisted of a Tektronix 5110 preamplifier (Tektronix Inc., Oregon, USA) connected to a digitizing table (DIGIDATA 1440 Interface Axon Instruments Inc., CA, USA). The electrical stimulus was generated by an electrical pulse unit (Cibertec CS-20). Axoscope 10.2 was used (Axon Instruments Inc, CA, USA) for data acquisition and analysis.

A pin recording electrode was inserted on the plantar pad of the treated leg and a pin reference electrode on the animal's back. Once the sciatic nerve was exposed, a monopolar needle electrode was used to apply a voltage-dependent stimulus at the origin of the nerve (preinjury area next to the spine, Figure 1(b)) and then the postinjury area (Figure 1(b)). The space between the pre and postinjury areas was 5 mm. In both cases, the voltage was increased from 0 Volts to a maximum value of the compound muscle action potentials (CMAPs). The duration of each stimulus was 50 *μ*s, and the average voltage required was 9 V. 10 records are made at 1 Hz. The variables studied were amplitude of the CMAPs measured between the maximum and minimum peak of the recording (expressed in millivolts (mV); latency, measured as the time elapsed from the stimulus artifact and the start of the electromyographic recording, expressed in milliseconds (ms); and ratio between postfibrosis latency and prefibrosis latency. This parameter is indicative of nerve conduction through the area of fibrosis, and a ratio of 1 means that the conduction is complete and the further away from a value of 1, the conduction is worse.

### 2.3. Ultrasonography

**Figure 2:**
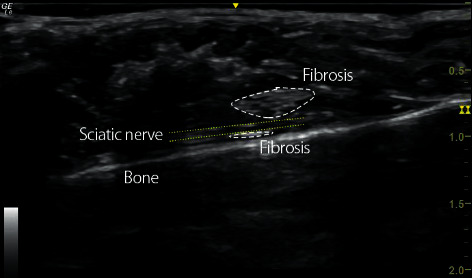
Nerve ultrasonography. Ultrasound imaging (longitudinal *B*-mode) shows sciatic nerve (yellow discontinue lines) with surrounding fibrosis (white discontinue lines).

Ultrasound is considered an optimal imaging technique to evaluate the disorders of peripheral nerves such as entrapment neuropathy. These include adjacent soft tissue masses (such as fibrosis, fluid collections, cysts, or tumor) [17]. Nerve ultrasonography was performed in mouse using an ultrasound device (General Electric, LOGIQ E R7) and a transducer (General Electric, L10-22-RS). In Figure 2, longitudinal images of the sciatic nerve show a hypoechoic tubular structure interspersed with hyperechoic lines representing the perineurium, and fibrosis is visualized as hyperechoic structure around the nerve.

### 2.4. Percutaneous Needle Electrolysis Application

During the PNE technique, the mouse was placed in the prone position with the hind legs extending away from the body (Figure 1(c)). The treatment protocol evaluated was 1.5 mA for 3 seconds and 3 repetitions (1.5/3/3) [16]. The equipment used to generate the galvanic current was Physio Invasiva® CE0120 (PRIM Physio; C/F *n*°15, Polígono Industrial *n*°1 – 28938, Móstoles, Spain). It produced galvanic current through the cathode (modified electrosurgical scalpel with the needle) while the anode (handheld electrode) is in contact with the mouse. An acupuncture needle 0.30 mm × 30 mm (Physio Invasiva® needles, PRIM Physio, Spain; uncoated steel needle with rigid metal handle with guide, Korean type) was used in all procedures.

Once the nerve conduction was recorded, PNE was applied according to the technique described by Valera-Garrido and Minaya-Muñoz [16]. The insertion in the fibrotic tissue was performed in two consecutive phases. At the initial phase, place the needle on the scar using a long-axis approach at a 30-degree angle in alignment with the nerve, positioning it without forcing, to perceive the resistance of the tissue. Then, the galvanic current was applied which allowed a slight advance of the needle. Five minutes after applying the percutaneous needle electrolysis, the protocol for determining nerve conduction was repeated.

### 2.5. Statistical Procedure

Values are expressed as mean ± SEM. Sometimes, the values are expressed as “Percentage of change.” This is defined as (experimental value/control value) × 100. We used the two-tailed Welch's *t*-test for unpaired values because our variances were not equal. We prefer this test as it is more conservative than the ordinary *t*-test. Differences were considered significant at *P* < 0.05.

## 3. Results

**Figure 3:**
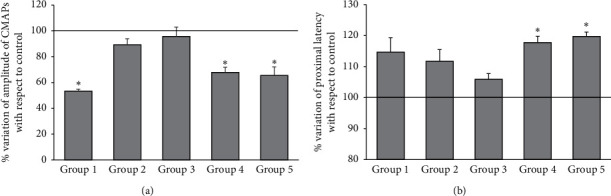
Neurography of the sciatic nerve. (a) Amplitude of the CMAPs stimulating the nerve in the preinjury area. Values expressed as % of variation of treated limb compared to the untreated leg (control). Group 1, BSA 35%–Glt 2% volume GG 50 *μ*l; *n* = 10 animals. Group 2, BSA 35%–Glt 2% volume GG 20 *μ*l; *n* = 10 animals. Group 3, BSA 35%–Glt 2% volume GG 10–10 *μ*l, 1 week; *n* = 6 animals. Group 4, BSA 35%–Glt 2% volume GG 10–10 *μ*l, 2 weeks; *n* = 10 animals. Group 5, BSA 35%–Glt 2% volume GG 10–10 *μ*l, 3 weeks; *n* = 10 animals. ^*∗*^*P* < 0.05, respect to the control limb. (b) Nerve latency stimulating the nerve in the preinjury area. Values expressed as % of variation of treated leg compared to untreated limb (control). Group 1, BSA 35%–Glt 2% volume GG 50 *μ*l; *n* = 10 animals. Group 2, BSA 35%–Glt 2% volume GG 20 *μ*l; *n* = 10 animals. Group 3, BSA 35%–Glt 2% volume GG 10–10 *μ*l, 1 week; *n* = 6 animals. Group 4, BSA 35%–Glt 2% volume GG 10–10 *μ*l, 2 weeks; *n* = 10 animals. Group 5, BSA 35%–Glt 2% volume GG 10–10 *μ*l, 3 weeks; *n* = 10 animals. ^*∗*^*P* < 0.05, compared to the control limb.

A model of perineural fibrosis of sciatic nerve of mice was developed using a solution of albumin and glutaraldehyde. By varying the volume administered and posttreatment times, five experimental groups were obtained. Group 1 (GG 50 *μ*l) coagulated the albumin very quickly from the moment the glutaraldehyde came into contact with the BSA. Finally, the fibrous reaction created unwanted adhesions between the skin and the buttocks. After two weeks, the treated leg showed an atrophy of the posterior muscle pack evident to the naked eye (Figure 1(d)) and a 21% decrease in leg weight compared to the control leg (*P* < 0.05, Table 1). This is indicative of very aggressive nerve compression. Stimulating the nerve in the preinjury area, the amplitude of the CMAPs (Figure 3(a)) presented a reduction of 46.7% (*P* < 0.05) without modifying the latency (Figure 3(b)) (% of variation: 14.6, *P* > 0.05 compared to the control leg).

A smaller volume (20 *μ*l) was used in group 2 than in group 1. As in group 1, a significant inflammatory response (hyperemia and edema) was observed two weeks after the procedure, although there were fewer adhesions between tissues. In this case, atrophy was also less visible and palpable, with a loss of muscle mass of 11.6% compared to the control (*P* < 0.05; Table 1). CMAPs amplitude (Figure 3(a)) did not significantly decrease (*P* > 0.05) and latency did not significantly increase (*P* > 0.05; Figure 3(b)).

In group 3 (BSA 35%–Glt 2% volume GG 10–10 *μ*l, 1 week), trying to overcome the technical difficulties of the previous groups, the decision was made to administer the solutions separately: a micropipette for BSA and another for GG, mixing the compounds in the nerve area where the fibrosis was intended to be generated. Indeed, undesirable adhesions were reduced. The weight of the muscle group of the treated leg did not change (0.5% variation, *P* > 0.05; Table 1). Neither did the amplitude of the CMAPs (Figure 3(a); % de variation: 4.3%; *P* > 0.05) or the latency (Figure 3(b); % of variation: 5.8, *P* > 0.05).

**Figure 4:**
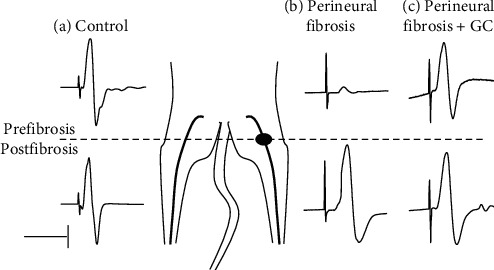
Raw data examples of nerve conduction. The records were made in the plantar muscle compartment of the left hind leg (control) and right hind leg (experimental). Fibrosis (black mole) was achieved with 10 *μ*l of GG, and recordings were made after 2 weeks. The sciatic nerve was electrically stimulated in the proximal portion of the fibrosis area (upper registers) and the distal portion to the fibrosis area (lower registers). Control recordings (*a*) were performed on portions of the sciatic nerve equivalent to those described above. The model recordings (*b*) were made 2 weeks after the creation of the perineural fibrosis. The nerve conduction recordings (*c*) were made 5 minutes after applying the galvanic current (1.5 mA for 3 seconds and 3 repetitions: 1.5/3/3). The records shown in (*a*), (*b*), and (*c*) correspond to different animals. The stimulus artifact has been modified for greater clarity. The dashed line shows the level of the fibrosis area. Vertical bar: 2 mV. Horizontal bar: 0.5 ms.

In group 4 (BSA 35%–Glt 2% volume GG 10–10 *μ*l), a marked decrease in the inflammatory reaction (mild hyperemia and edema) was observed after two weeks. A loss of muscle mass of only 14.7% (*P* < 0.05) was observed (Table 1). In addition, the amplitude of the CMAPs decreased very sharply (32.2%, *P* < 0.05; Figure 3(a)) and the proximal latency increased (17.7%; *P* < 0.05; Figure 3(b)).  Figure 4(b) shows a record of nerve conduction two weeks after triggering the perineural reaction in a group 4 mouse. Note how the fibrous reaction reduces the amplitude of the proximal to distal recording, indicating a partial block of nerve conduction.

For group 5 (BSA 35%–Glt 2% volume GG 10–10 *μ*l), the evaluation at three weeks no longer shows an inflammatory response (no hyperemia or edema), and there are very few adhesions. There was no variation in leg weight (% variation: 5.4%; *P* > 0.05; Table 1). The amplitude of the CNAPs (Figure 3(a)) was decreased by 34.6% (*P* < 0.05), and the latency (Figure 3(b)) was increased at 19.7% (*P* < 0.05).

### 3.1. Application of Percutaneous Needle Electrolysis in the Perineural Fibrosis Model

Based on the results obtained in the neurographies, the protocol used in group 4 (GG 10 *μ*l and evaluation at 2 weeks) was chosen for the development of the model. Initially, the treatment with percutaneous needle electrolysis was evaluated immediately after application.  Table 2 displays the values obtained in the pre and postfibrosis areas before and after applying electrolysis. A decrease in latency of 16% was observed in the prefibrosis area and 6% in the postfibrosis area. Moreover, an increase in the amplitude (mV) of the CMAPs of 48.7% (*P* < 0.05) was observed in the prefibrosis area without a significant variation in the postinjury area (Table 2). This means that the speed of nerve conduction increases. In no case have adverse or unwanted effects been observed.

Regarding the ratio between the pre and postfibrosis values to determine the ratio of the entire nerve segment with fibrosis, this parameter is indicative of nerve conduction recovery, since a ratio of 1 means that conduction is complete and the further away from a value of 1, the worse the conduction is.  Table 2 shows that this parameter is closer to 1 (0.99) as soon as PNE is applied.

In order to rule out the possibility of a relapse in the treatment using PNE, several neurographs were taken (*n* = 10) after 3 weeks. On this occasion, perineural fibrosis was only provoked in the left legs. Thus, the PNE treatment was applied to both the left (experiment) and right (control) legs. The legs had recovered their normal aspect and weight (% variation: 95.30 ± 1.49; *n* = 10, *P* > 0.05 compared to the control leg), and no signs of atrophy were observed. When making the incision to perform nerve conduction records, some scar remains were observed in the vicinity of the sciatic nerve. The nerve conduction achieved after treatment with percutaneous needle electrolysis was maintained at control values. Thus, for example, the amplitude of the CMAPs was similar to that of the control leg (% variation: 94.64 ± 1.66, *n* = 3, *P* > 0.05 compared to the control leg). The ratio between pre and postfibrosis values remained close to 1 (0.98 ± 0.03; *n* = 10, *P* > 0.05 compared to the control leg). This means that after three weeks, a greater reversal is obtained than that immediately achieved with PNE treatment. No adverse or undesirable effects were observed.

## 4. Discussion

Following the protocol previously described in rats by Lemke et al. [13], we were able to reproduce the animal model with perineural fibrosis in the sciatic nerve in a mouse. In the present study, the animal model has presented fibrotic perineural tissue and changes in the electrophysiological records of CMAPs and latency. Besides, macroscopic changes have been observed, such as initial inflammatory reaction and adhesions between adjacent tissues, generating fibrotic tissue around the sciatic nerve. As a consequence of the fibrosis, the treated leg has presented an atrophy and decrease in weight of the posterior muscles, as well as alterations in the compound action potential parameters of the nerve and latency. The authors [13] also highlight the limitation in being able to transfer the results observed in the animal model with perineural fibrosis to those observed in the human nerve, mainly due to the high regenerative response and adaptability presented by rats, a fact that we also believe occurs in the mouse, given the recovery of the weight of the posterior muscles, observed in group 5 (3 weeks) of our model.

The physiopathology of compression of the peripheral nerve involves an increase in pressure on the nerve [3]. This situation triggers a cascade of events. The increased pressure will result in venous stasis, increased vascular permeability, followed by edema and fibrosis [3]. In addition, an increase in connective tissue will occur, visible in the thickening of the epineurium and perineurium. The chronic increase in pressure on the nerve also produces an alteration in the endoneural circulation [6] and in the myelin sheaths, resulting in focal demyelination and subsequent axonal damage [5]. Somehow, the application of percutaneous needle electrolysis used in this study addresses this cascade of events. The mechanical effect caused by the needle on the tissue surrounding the nerve seems easy to justify, since the fact of introducing an element may partially cut or remove tissue. Petrover and Richette [18] by performing a minimally invasive approach, combining the use of ultrasound and mechanical surgery techniques, describe releasing the median nerve. These authors point out that it is possible to release the median nerve without completely cutting the flexor retinaculum, only cutting deeper parts close to the nerve. The needles used in the present study to apply GC are completely blunt [19], and its mechanical action can hardly perform such a selective function. Moreover, percutaneous needle electrolysis seems to have an immediate direct fibrinolytic action, thus freeing the nerve from its entrapment [16, 20].

Currently, electrical stimulation techniques are being used in the treatment of Raynaud's disease since the application of a galvanic electrical current produces a vasodilatory effect on blood flow (for example, [21]). If nerve entrapment triggers a cascade of events involving vascular involvement, percutaneous needle electrolysis may solve this problem by partially justifying the rapid reversal of nerve conduction when applied.

In addition, several studies have reported that cells can respond directionally to electrical fields applied in both in vitro and in vivo environments, via a phenomenon called electrotaxis (reviewed by [22]). Endothelial electrotaxis is also involved in angiogenesis [23], and the application of percutaneous electrical currents can affect the inflammatory mediators in the damaged tissue and promote a new vascularization of the injured area [24]. Therefore, both angiogenesis and electrotaxis of inflammatory cells can be beneficial in the treatment of entrapment neuropathies. This would justify the progress over time of the changes produced by GC.

In summary, the release of the sciatic nerve from its entrapment by the action of PNE offers several pathophysiological explanations: (a) a mechanical action of the needle used as electrode is not discarded but unlikely; (b) immediate vasodilation to the GC treatment would justify the rapid recovery of nerve conduction; (c) an immediate fibrolytic action of GC on the fibrous reaction artificially induced to trap the nerve is also not discarded; and (d) angiogenesis and electrotaxis of inflammatory cells would help justify the improvement of GC-induced changes over time.

## 5. Conclusions

This study has obtained an easily reproducible model of sciatic nerve entrapment in mice. This model can be extrapolated to nerve entrapments in humans since it presents the clinical signs of muscular atrophy, decrease of the compound muscle action potential, and slowing of the nerve conduction. All these signs are reversed by means of nerve release applying percutaneous needle electrolysis (PNE). A partial reversal is obtained immediately, but after three weeks, we obtained values identical to the controls. Furthermore, no undesirable effects were observed.

This technique can be extrapolated to nerve entrapments in patients, for example, in CTS, by releasing the median nerve, in a simple, fast, safe, effective way and with fewer adverse effects than surgical treatment.

## Figures and Tables

**Table 1:** Leg weights with perineural fibrosis of the sciatic nerve.

Group	1	2	3	4	5
% variation of the weight of the treated leg respect to the control leg	78.6 ± 1.3^*∗*^	88.3 ± 1.0^*∗*^	100.5 ± 1.2	85.2 ± 1.4^*∗*^	94.5 ± 1.9

Group 1: GG 50 *μ*l was administered and evaluated at 2 weeks. Group 2: GG 20 *μ*l was administered and evaluated after 2 weeks. Group 3: GG 10 *μ*l was administered and evaluated 1 week later. Group 4: GG 10 *μ*l was administered and evaluated at 2 weeks. Group 5: GG 10 *μ*l was administered and evaluated at 3 weeks. Values are expressed as mean ± SEM. ^*∗*^*P* < 0.05, treated leg values with respect to control leg values. *N* = 10 control legs and 10 experiment legs from 10 mice.

**Table 2:** Neurography of the sciatic nerve treated with GC.

		Before GC	After GC
Peak-to-peak amplitude (mv)	Postfibrosis	7.19 ± 0.91	10.90 ± 0.61^*∗*^
	Prefibrosis	0.83 ± 0.84	8.60 ± 1.02
	Ratio post/prefibrosis	1.41 ± 0.29	0.79 ± 0.09^*∗*^

Latency (ms)	Postfibrosis	0.174 ± 0.004	0.151 ± 0.007^*∗*^
	Prefibrosis	0.155 ± 0.007	0.153 ± 0.008
	Ratio post/prefibrosis	0.88 ± 0.02	0.99 ± 0.04^*∗*^

GC, galvanic current (1.5/3/3). Values are expressed as mean ± SEM. ^*∗*^*P* < 0.05 respect to the values obtained before applying the GC. *N* = 20 sciatic nerves from 10 mice.

## Data Availability

The data used to support the findings of this study are included within the article and supplementary materials.

## Supplementary Materials

Supplementary Materials The supplementary file contains the data referred in Figures 3(a) and 3(b).
