# Supplementary material for: Percutaneous Needle Electrolysis Reverses Neurographic Signs of Nerve Entrapment by Induced Fibrosis in Mice
Source: Evid Based Complement Alternat Med. 2020 Dec 22;2020:6615563. doi: 10.1155/2020/6615563 (PMC7772050; doi:10.1155/2020/6615563)
Supplement: Supplementary Materials — The supplementary file contains the data referred in Figures 3(a) and 3(b). [file 6615563.f1.pdf]

FIGURE 3A

% variation of amplitude of CMAPs with respect to control

	MEAN	SEM	N
**Group 1**	53,30	1,49	10
**Group 2**	89,20	4,68	10
**Group 3**	95,70	7,18	10
**Group 4**	67,80	3,98	10
**Group 5**	65,40	6,68	10

FIGURE 3B

% variation of proximal latency respect to control

	MEAN	SEM	N
**Group 1**	114,67	4,65	10
**Group 2**	111,75	3,73	10
**Group 3**	105,90	1,87	10
**Group 4**	117,75	2,02	10
**Group 5**	119,75	1,36	10
